# Analysis on morphological characteristics and identification of candidate genes during the flowering development of alfalfa

**DOI:** 10.3389/fpls.2024.1426838

**Published:** 2024-08-13

**Authors:** Fenqi Chen, Kuiju Niu, Huiling Ma

**Affiliations:** ^1^ College of Pratacultural Science, Gansu Agricultural University, Lanzhou, Gansu, China; ^2^ Key Laboratory of Grassland Ecosystem, Ministry of Education, Pratacultural Engineering Laboratory of Gansu Province, Lanzhou, Gansu, China; ^3^ Sino-U.S. Center for Grazingland Ecosystem Sustainability, Lanzhou, Gansu, China

**Keywords:** alfalfa, flower development, oxidation-reduction, hormone signal transduction, transcription factors

## Abstract

Flower development is a crucial and complex process in the reproductive stage of plants, which involves the interaction of multiple endogenous signals and environmental factors. However, regulatory mechanism of flower development was unknown in alfalfa (*Medicago sativa*). In this study, the three stages of flower development of ‘*M. sativa* cv. Gannong No. 5’ (G5) and its early flowering and multi flowering mutant (MG5) were comparatively analyzed by transcriptomics. The results showed that compared with late bud stage (S1), 14287 and 8351 differentially expressed genes (DEGs) were identified at early flower stage (S2) in G5 and MG5, and 19941 and 19469 DEGs were identified at late flower stage (S3). Compared with S2, 9574 and 10870 DEGs were identified at S3 in G5 and MG5, respectively. Venn analysis revealed that 547 DEGs were identified among the three comparison groups. KEGG pathway enrichment analysis showed that these genes were involved in the development of alfalfa flowers through redox pathways and plant hormone signaling pathways. Key candidate genes including *SnRK2*, *BSK*, *GID1*, *DELLA* and *CRE1*, for regulating the development from buds to mature flowers in alfalfa were screened. In addition, differential expression of transcription factors such as MYB, AP2, bHLH, C2C2, MADS-box, NAC, bZIP, B3 and AUX/IAA also played an important role in this process. The results laid a theoretical foundation for studying the molecular mechanisms of the development process from buds to mature flowers in alfalfa.

## 1 Introduction

The flower development of crops is closely related to yield and quality, and is important agronomic trait, which also is most important developmental event in the life cycle of higher plants (Wang and Li, 2008; Leijten et al., 2018). Flower development is an important physiological process for the seed formation and development of plant species, and this process is finely regulated by both endogenous plant signals and external environmental factors (Leijten et al., 2018). In major crops, it has been reported that flower development is the basis for seed yield and quality formation, directly determining the economic benefits of crops (Zhang et al., 2022). Therefore, the molecular mechanism of flower trait regulation has always been a hot and difficult topic in plant developmental biology research. Especially in *Arabidopsis*, significant progress has been made in the genetic pathways involved in flower development (Jack, 2004; Andrés and Coupland, 2012). Plant developmental biologists have identified several key genes involved in flower development in *Arabidopsis*. Based on similarity searches, it has been found that many of these genes identified in *Arabidopsis* are conserved in legume species such as soybean (*Glycine max)*, *Medicago truncatula*, and lotus (*Nelumbo nucifera*) (Hecht et al., 2005). However, a precise sequence of events governing flower development in legume plants has yet to be studied. Due to the distinctive floral morphology in legumes, researchers speculate that their flower development may not adhere to the classical ABC model (Singh et al., 2013). In *Arabidopsis*, organ development is sequential without overlap in initiation times, driven by a set of genes determining the onset of each organ. Conversely, in legume plants, multiple floral organs can initiate simultaneously, a phenomenon supported by previous studies, which may be influenced by the unique floral morphology within the Papilionoideae subfamily. Singh et al. (2013) reported overlapping expression of stage-specific genes during chickpea flower development, suggesting this may underlie the simultaneous initiation and development of different whorls. However, research on flower development in legume plants remains limited, and this hypothesis has yet to be substantiated over the past decade. Therefore, it is essential to elucidate the molecular basis of flower development in legume plants, which could provide valuable insights into the molecular mechanisms involved in flower development across other crops, and offer potential genetic targets for crop improvement through genetic engineering and modern molecular editing technologies.

Analyze the flower transcriptomes of model plants *Arabidopsis thaliana* and rice (*Oryza sativa*) at different developmental stages could identify numerous genes involved in flower development (Hennig et al., 2004; Wellmer et al., 2006). Identification of candidate genes expressed during rose (*Rosa rugosa*) development of flower using cDNA microarrays (Dubois et al., 2011). Similarly, the use of cDNA amplification fragment length polymorphism analysis and proteomics techniques has revealed many comprehensive molecular genetic events related to flower development (Zhang et al., 2013). In recent years, deep sequencing has been widely used to obtain a global view of transcriptome dynamics during developmental processes, such as flower development (Singh et al., 2013). In addition, RNA-Seq analysis of *Dendrocalamus latiflorus* and soybean revealed significant events that occurred during the floral transition process (Wong et al., 2009; Zhang et al., 2012). Transcriptome sequencing is a convenient tool for studying gene expression and determining inferred gene functions (Wang et al., 2009; Ozsolak and Milos, 2011; Jain, 2012). Many studies have demonstrated the role of RNA-seq in various biological environments (Weber et al., 2007; Zenoni et al., 2010; Li et al., 2010b; Yang et al., 2011). Although rapid progress has been made in understanding the transcriptional programs of several plant flower development processes using RNA-seq technology, there have been relatively few studies conducted in leguminous plants (Benedito et al., 2008; Libault et al., 2009; Severin et al., 2010). Considering the morphological specificity of different plant lineages, studying the gene expression profile during flower development in leguminous plants such as alfalfa (*Medicago sativa*) is of great significance.

Alfalfa as a perennial high-quality leguminous grass, known as the “king of forage”, has high grass yield and rich protein content, which plays an irreplaceable role in the healthy development of dairy industry and other grass husbandry (Liu et al., 2017; Dong et al., 2021). Alfalfa is widely cultivated in North America, Asia, and other continents and is one of the most economically valuable crops in the world (Liu et al., 2013, 2017). Alfalfa is the fourth-largest growing crop in the United States after wheat, corn, and soybean (Chen et al., 2020b). In China, the alfalfa planting area is mainly distributed in the 14 provinces in the northern region, and it is also one of the most widely planted grasses (Dong et al., 2021). In addition to feed quality, other agronomic traits of alfalfa are also very ideal, including adaptability to different environments, abundant biomass yield, drought resistance, and more importantly, nitrogen fixation ability through symbiosis with rhizobia (Radovic et al., 2009; Lei et al., 2017; Singer et al., 2018). The latter accounts for the majority of alfalfa’s nitrogen demand and ensures high protein levels in the leaves. As a deep-rooted plant, alfalfa has high water use efficiency and nitrogen fixation ability, which are excellent qualities for sustainable agriculture (Kulkarni et al., 2018). Therefore, as one of the four major crops in the world, the position of alfalfa in the development of agriculture is beyond doubt. At present, only a small number of studies have verified the function of flower regulatory factors in alfalfa. However, there have been no systematic studies on the unique regulation genes of flower development in alfalfa. The availability of RNA-seq technology also provides excellent opportunities for conducting such research in leguminous plants. Therefore, G5 and its early flowering and multi flowering MG5 were selected as experimental materials in this study to observe the morphological characteristics of flower development, and its transition from bud to mature flower. On this basis, we selected three key stages of flower development in two materials for transcriptome sequencing to obtain gene expression trends, which can be used for in-depth research on key genes controlling flower development in alfalfa. This has important theoretical value and practical significance for elucidating the regulatory network of flower development in leguminous plants, cultivating high-quality leguminous crops with more suitable inflorescence morphology to increase seed yield.

## 2 Materials and methods

### 2.1 Experimental materials

*Medicago sativa* cv. Gannong No. 5 (G5) and its natural mutant MG5 were both provided by Gansu Chuanglv Grassland Technology Co., Ltd. In 2018, G5 was planted using single-seed sowing by our research group in Nanhua Town, Gaotai County, Gansu Province, China (longitude 99° 47 ′ E, latitude 39° 18 ′ N). After planting G5, observe its various growth indicators annually. In 2021, a naturally mutated plant was discovered and identified, with a flowering period 10 days earlier than G5 and typical characteristics of low first flower nodes and multiple inflorescences. It was labeled as MG5 and asexual propagation was carried out using cutting techniques to expand the MG5 population. Simply put, the MG5 branches at the beginning of the flowering period were selected, and cut into cuttings using scissors, with each cutting being approximately 5 cm long and only containing one node. The leaves were removed from each node without damaging the leaf buds. The prepared cuttings were then planted obliquely in the field for cultivation, allowing them to root and grow into individual alfalfa plants. After further detailed identification, we found that the newly propagated MG5 population retains all the characteristics of the mother plant. To ensure simultaneous growth cycles of G5 and MG5, a G5 material was also chosen for asexual reproduction and propagation alongside the asexual reproduction of MG5.

### 2.2 Determination of morphology and endogenous hormone content

In September 2023, we selected G5 and MG5 as research materials and conducted statistical analysis on their inflorescence morphology indicators such as the number of primary branches per plant, the number of flowers per branch, the number of small flowers per branch, the number of seeds contained in each pod, and the pod setting rate. Each indicator was repeated 10 times, with each repetition being the measurement of a single plant. And the samples were taken from flower buds at the late bud stage (S1) and florets at the early flower stage (S2) and late flower stage (S3), mainly from the apical inflorescence of a single branch, and do not contain the inflorescence axis. Three biological replicates were obtained, each from a different individual plant. The samples were quickly stored in liquid nitrogen and then frozen in an ultra-low temperature freezer (-80 °C) for subsequent analysis. The relative contents of zeatin (ZT), 3-Indoleacetic acid (IAA), salicylic acid (SA), abscisic acid (ABA) and gibberellin A3 (GA_3_) were determined in the inflorescence tissues of alfalfa at three stages of flower development by high performance liquid chromatography (HPLC) as described according to Zhao et al. (2021).

### 2.3 Transcriptional analysis

#### 2.3.1 Total RNA extraction and library construction

Separate and purify RNA from individual buds or florets of G5 and MG5 materials at three stages according to the protocol provided by TRIzol (Invitrogen, CA, USA) reagent manufacturers. Then, the quantity and purity of total RNA extracted from each flower sample were controlled using NanoDrop ND-1000 (NanoDrop, Wilmington, DE, USA) for quality control. The total RNA of the quality-controlled samples was used for the construction of the RNA-seq library, which was completed by Biomarker Technologies Co., Ltd. (Beijing, China). The mRNA with PolyA was specifically captured by two rounds of purification using oligo (dT) magnetic beads (25-61005, Thermo Fisher, USA). The captured mRNA was fragmented under high temperature conditions and treated at 94°C for five to seven min. The fragmented RNA was used to synthesize cDNA. Then, Escherichia coli DNA polymerase I (New England Biolabs, Ipswich, MA, USA [NEB]) and RNase H (NEB) were used for double strand synthesis. These complex double strands of DNA and RNA were transformed into DNA double strand, dUTP solution (Thermo Fisher, USA) was incorporated into the double strand, and the ends of the double stranded DNA were complemented to the flat ends. An A-base was then added to each end to enable it to ligate with a connector with a T-base at the end, and the fragment size was then screened and purified using magnetic beads. The second strand was digested with UDG enzyme (NEB), and then the library with a fragment size of 300 bp ± 50 bp was formed by PCR - pre-denaturation held at 95°C for three min, denaturation at 98°C for a total of eight cycles of 15 seconds each, annealing to 60°C held for 15 seconds, extension at 72°C for 30 seconds, and final extension held at 72°C for 5 minutes. Finally, it was double-end sequenced using illumina Novaseq™ 6000 in PE150 sequencing mode according to standard practice. The original sequencing reads have been submitted to the SRA at NCBI (Accession number: PRJNA1100750).

#### 2.3.2 Quality assessment of sequencing results

Low quality and duplicate sequences were removed from the raw reads to obtain clean data, and then the clean reads were compared with the fourth version of the *Medicago sativa* reference genome (version 3) (https://figshare.com/articles/dataset/genome_fasta_sequence_and_annotation_files/12327602) using the HISAT2 (Hisat2-2.0.4) software (Kim et al., 2015). Finally, RSEM software was used to detect the expression level of the genes (Li and Dewey, 2011).

#### 2.3.3 Analysis and identifiction of DEGs

Genes were analyzed for significant differences between the comparison groups using the R package DESeq2 (Pertea et al., 2015), and genes with differential |Fold change (FC)| ≥ 2 and FDR<0.01 were defined as DEGs for screening. The screened DEGs were analyzed for GO (Gene Ontology) and KEGG (Kyoto Encyclopedia of Genes and Genomes) enrichment.

### 2.4 qRT-PCR of DEGs

To improve the accuracy and reliability of the experimental results, we performed quantitative real-time PCR (qRT-PCR) on the total RNA used for library construction. The RNA was reverse transcribed for cDNA synthesis with PrimeScript™ RT reagent Kit with gDNA Eraser (TAKARA, Japan) according to the instructions provided by the manufacturer. 12 DEGs were randomly selected, and specific primers were designed by Primer-BLAST on NCBI (https://www.ncbi.nlm.nih.gov/tools/primer-blast) (**Supplementary Table S1**). qRT-PCR amplification was performed on LightCycler 96 (Roche, Basel, Switzerland) using super real premix plus (SYBR Green) (Tiangen, Shanghai, China). Each treatment had three biological replicates and each real time PCR was performed at 20 µL. The reaction volume included 10 µL Superreal PreMix Plus, 3.4 µL ddH_2_O, 0.8 µL forward primer (10 µ mol·L^-1^), 0.8 µL reverse primer, and 5 µL template cDNA. The amplification process followed the procedure of Chen et al. (2020a). The relative transcription levels of the selected genes were calculated with the 2^−ΔΔCT^ method and normalized to the expression levels of the *Actin* gene (AES78237. 1).

### 2.5 Statistical analysis of data

The above experimental data were statistically plotted by Microsoft Excel 2019, and the one-way ANOVA (P < 0.05) and Paired samples t-test (P < 0.05) and (P < 0.01) was analyzed by IBM SPSS 24.0 software.

## 3 Results and analysis

### 3.1 Analysis of morphological differences between G5 and MG5

**Figure 1:**
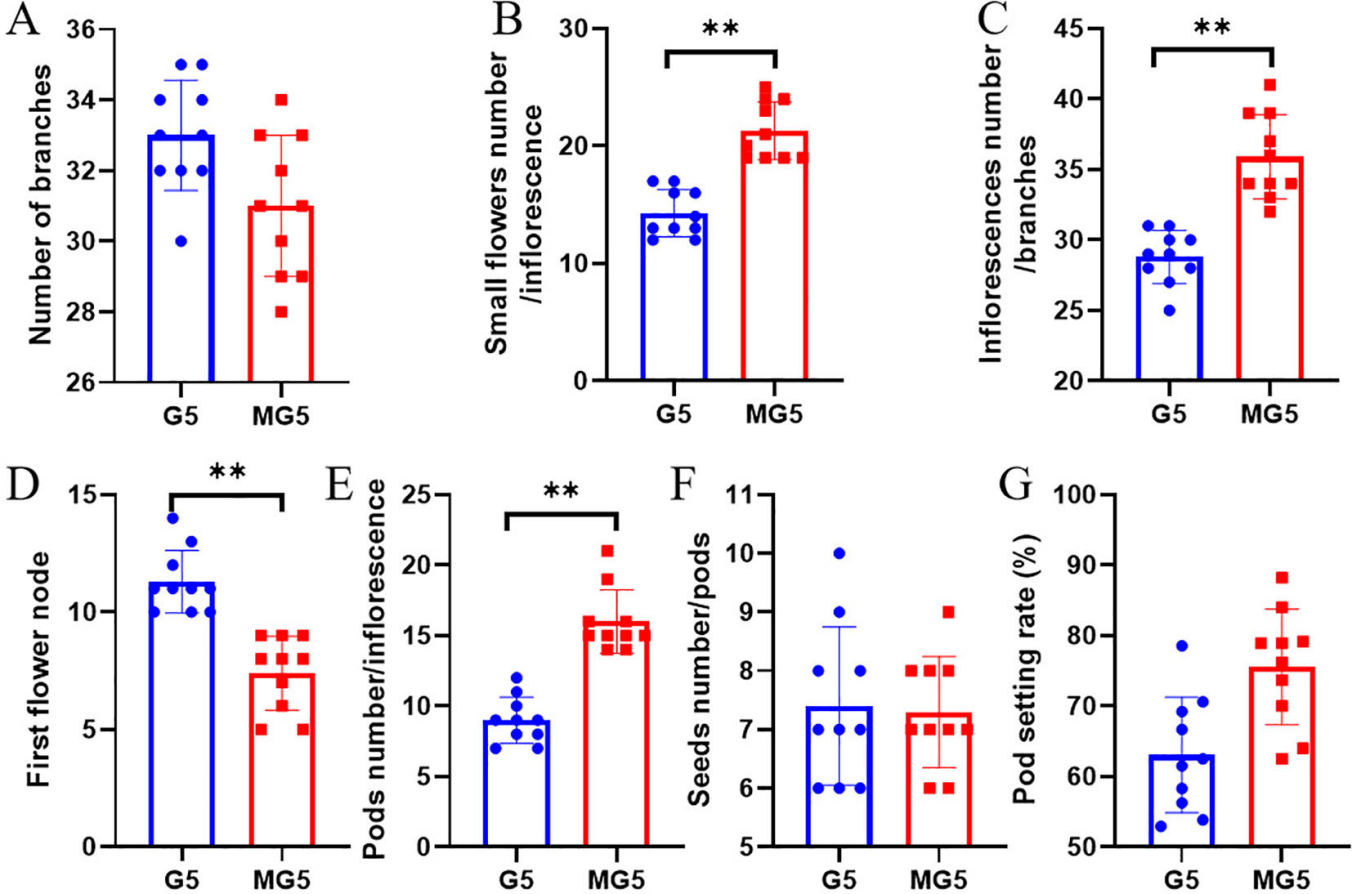
Analysis of morphological differences between G5 and MG5. Paired sample t-tests were conducted between different materials for each indicator, where * represents the P<0.05 level and ** represents the P<0.01 level. **(A)** Number of branches; **(B) **small flowers number / inflorescence; **(C)** inflorescence number / branch; **(D) **First flower node;**(E) **Pods number / inflorescence;**(F)** Seeds number / pods; **(G)** Pod setting rate.

Through statistical analysis of the morphological indicators of G5 and MG5, it was found that there was no significant difference in the number of branches per plant of naturally mutated alfalfa MG5 compared to G5 (P>0.05) (**Figure 1A**). However, compared with G5, the number of small flowers per inflorescence and the number of inflorescences per branch of MG5 were significantly increased (P<0.01), with increases of 48.95% and 24.65% respectively (**Figures 1B, C**); The position of the first flower node of MG5 also significantly decreased by 34.51% (**Figure 1D**); The number of pods per inflorescence also significantly increased, reaching 77.78% (**Figure 1E**). Compared with G5, there was no significant difference in the number of seeds/pods and pod setting rate (**Figures 1F, G**). This fully demonstrated that MG5 showed significant differences from G5 at the flowering time and the number of floral organs, so we chose three typical flowering stages from these two materials for further analysis to better identify the genes involved in flower development process.

### 3.2 Analysis of endogenous hormone differences between G5 and MG5 during flowering period

**Figure 2:**
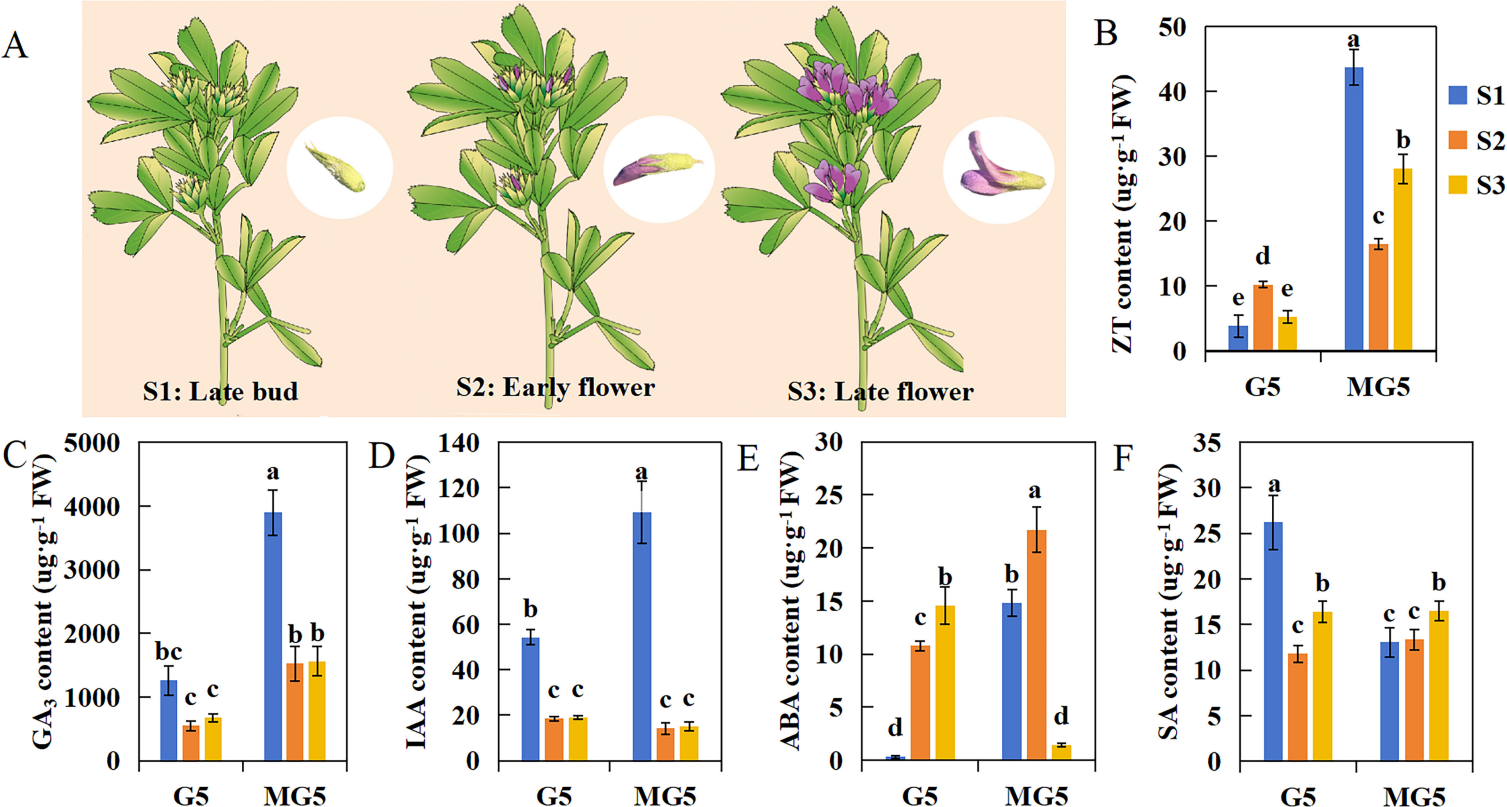
The difference analysis of endogenous hormone content between G5 and MG5 at three stages.**(A)** A schematic diagram of the three developmental stages of alfalfa;**(B) **ZT, zeatin; **(C) **GA3, gibberellin; **(D)** IAA, 3-indoleacetic acid;**(E) **ABA, academic acid; **(F)** SA, salicylic acid. One-way ANOVA was conducted on endogenous hormones from different materials and stages, with different lowercase letters indicating differences at the P<0.05 level.

We selected three stages of flowering: buds at the late bud stage (S1: sepals tightly enclosing the entire small flower, with overall green appearance) and florets at the early flower stage (S2: flowers extending beyond sepals, keel, banner, and wings still closely fused together, half of the flower displaying purple coloration and the remaining half green) and late flower stage (S3: green sepals occupying only one-third of the total flower length, banner petal opening at approximately 45 degrees, wings clearly observable) (**Figure 2A**). And the contents of endogenous hormones including ZT, GA_3_, IAA, ABA, and SA were analyzed at these three stages. We found that the contents of ZT and GA_3_ in MG5 were significantly higher than that in G5 during the S1, S2, and S3 stages (P<0.05) (**Figures 2B, C**); The contents of IAA and SA were significantly different only at S1, specifically in MG5, where IAA content was significantly higher than G5, and SA content was significantly higher in G5 than MG5 (**Figures 2D, F**); The content of ABA was significantly higher in the first two stages of MG5 (S1 and S2) than in G5, but lower at S3 than in G5 (**Figure 2E**). In addition, the contents of GA_3_ and IAA showed similar trends in the three stages of the two materials, while the contents of ZT and ABA peaked at the S1 and S2 stages of MG5, both significantly higher than G5 (**Figures 2B, E**). The SA content was opposite at S1, showing an early peak at the S1 stage of G5 (**Figure 2F**). The changes in endogenous hormone content were often regulated by the expression of many genes. Therefore, we speculated that the premature peak of endogenous ZT and ABA contents in MG5 compared to G5 may be one of the reasons for its high number of flower orders and small flowers, while the high SA content in G5 during the S1 stage may be a factor leading to its lower number of flower orders and small flowers than MG5.

### 3.3 RNA sequencing analysis

RNA-seq sequencing obtained a total of 118.69 Gb of Clean Data from 18 alfalfa flower samples, with all samples achieving 5.91 Gb of Clean Data (**Supplementary Table S2**). The Q30 value range of filtered clean data was 95.16 to 97.13%, with an average value of 95.90%; The GC content ranges from 41.43% to 42.94%, with an average of 42.03%. Compared with the reference genome of homologous tetraploid alfalfa (**Supplementary Table S3**), we obtained 31,207,730 to 41,989,022 alignment sequences, with an average of 38,482,070; The comparison rate was 78.95 to 91.06%, with an average of 87.11%. Based on the comparison results, 23,897 new genes were identified, among which, 12,564 genes identified have complete functional annotations. These data indicate that the data obtained from the transcriptome was sufficient for the next analysis.

### 3.4 Comparative analysis of DEGs in three flower stages

**Figure 3:**
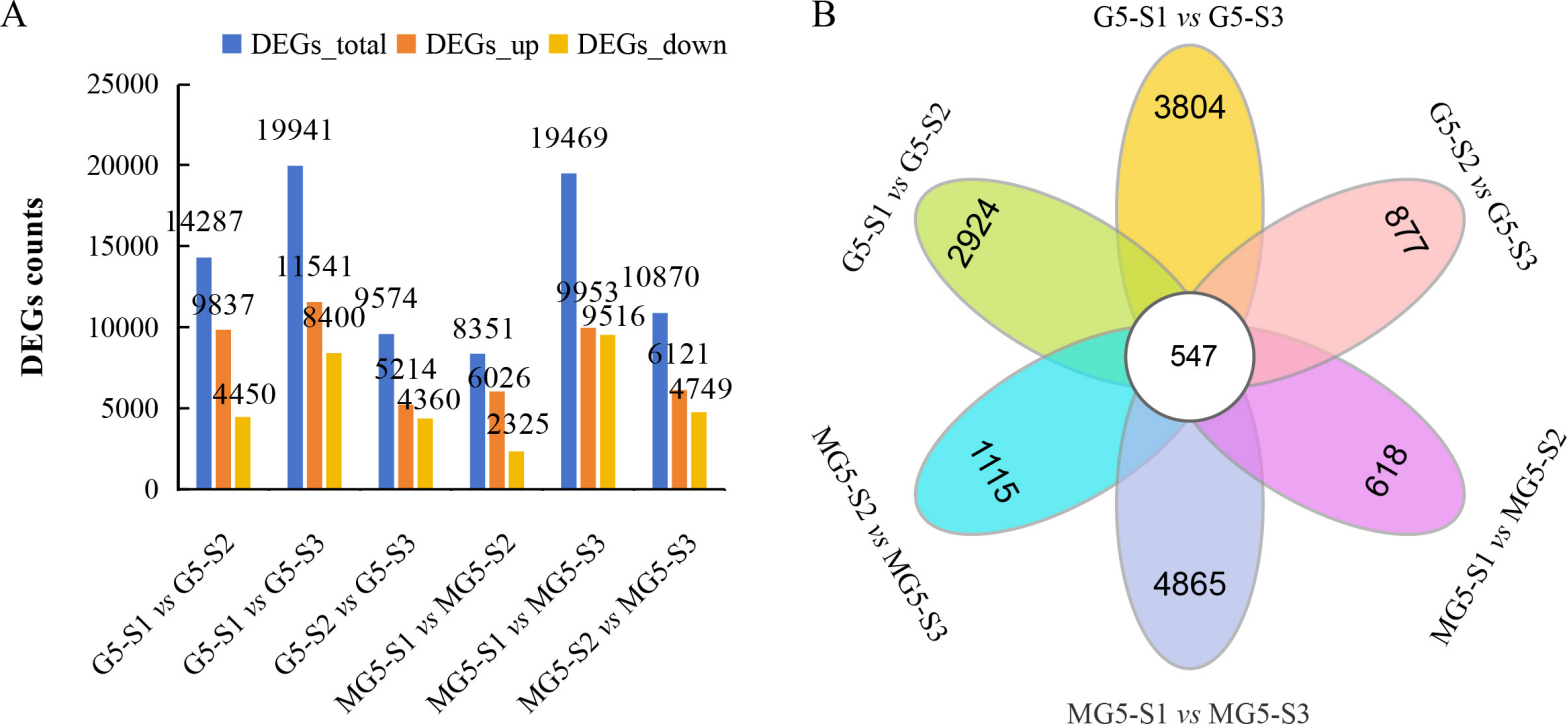
Comparative analysis of DEGs between G5 and MG5 during three stages. **(A)** The distribution of DEGs in the different comparison groups of G5 and MG5.; **(B)** Venn diagram analysis shows differences in DEGs during three stages.

Based on the criteria of |FC|≥2 and FDR<0.01 as the selection standards for DEGs, 14287 DEGs (9837 up-regulated and 4450 down-regulated) in the comparison group G5-S1 *vs* G5-S2, 19941 DEGs (11541 up-regulated and 8400 down-regulated) in G5-S1 *vs* G5-S3, 9574 DEGs (5214 up-regulated and 4360 down-regulated) in G5-S2 *vs* G5-S3, 8351 DEGs (6026 up-regulated and 2325 down-regulated) in MG5-S1 *vs* MG5-S2, 19469 DEGs (9953 up-regulated and 9516 down-regulated) in MG5-S1 *vs* MG5-S3, and 10870 DEGs (6121 up-regulated and 4749 down-regulated) in MG5-S2 *vs* MG5-S3 were identified, respectively (**Figure 3A**; **Supplementary Table S4**). Compared to G5, MG5 showed fewer total DEGs and fewer up-regulated DEGs in the S1 *vs* S2 and S1 *vs* S3 comparisons, indicating different response patterns in alfalfa flower development at the transcriptomic level. To analyze specifically expressed genes across the three stages and visually illustrate shared and unique DEGs between the two materials in each comparison group, Venn diagram analysis was performed, which identified 547 DEGs across six comparison groups (**Figure 3B**). Specifically, 2924 unique DEGs in G5-S1 *vs* G5-S2, 3084 unique DEGs in G5-S1 *vs* G5-S3, 877 unique DEGs in G5-S2 *vs* G5-S3, 618 unique DEGs in MG5-S1 *vs* MG5-S2, 4865 unique DEGs in MG5-S1 *vs* MG5-S3, and 1115 unique DEGs in MG5-S2 *vs* MG5-S3 were identified, respectively, which demonstrated that there was distinct transcriptional differences between MG5 and G5 across the three stages of flower development.

### 3.5 Functional enrichment analysis of DEGs

#### 3.5.1 GO enrichment analysis

**Figure 4:**
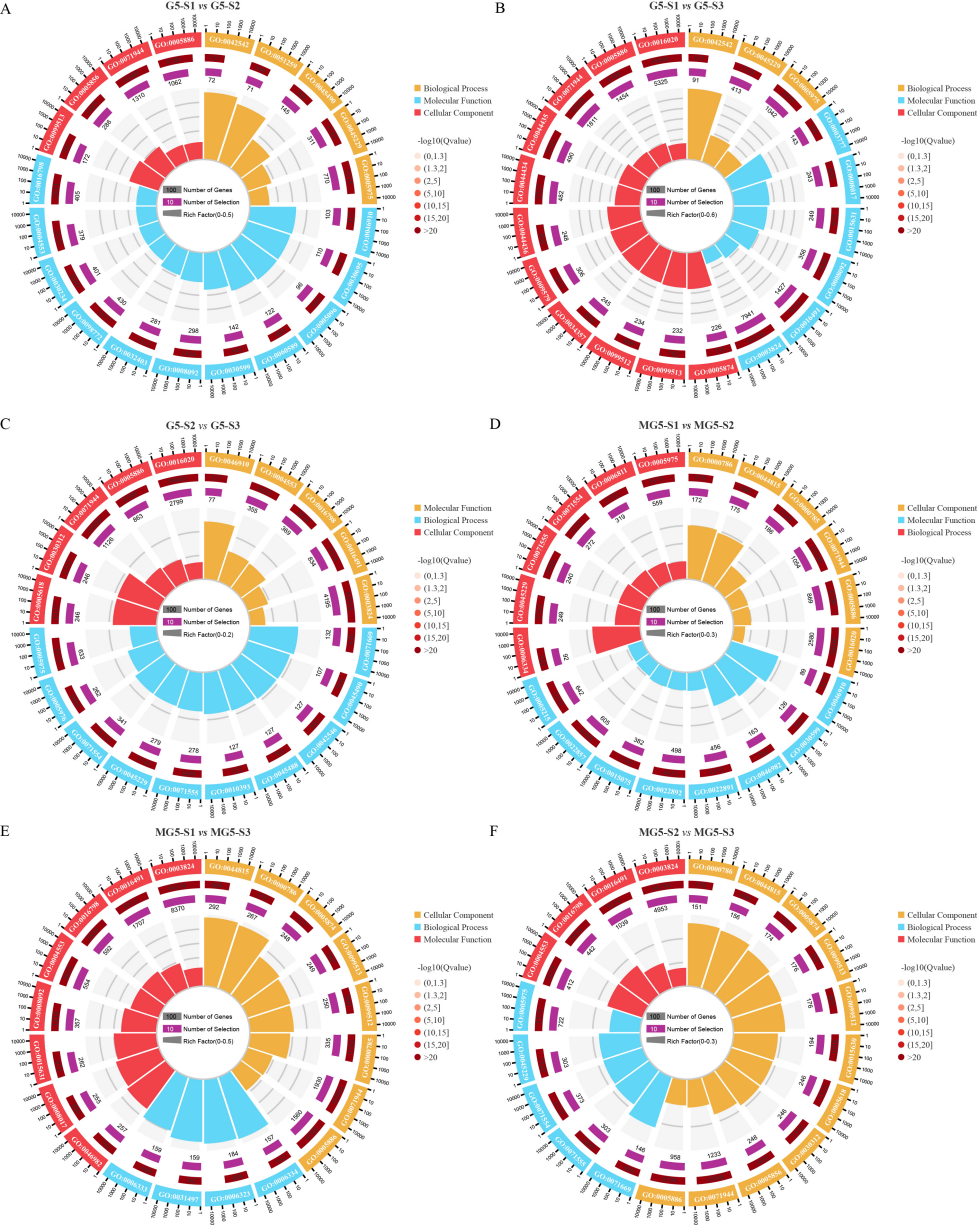
Gene ontology (GO) enrichment analysis of the DEGs between MG5 and G5. **(A)** G5-S1 vs G5-S2; **(B)** G5-S1 vs G5-S3;**(C)** G5-S2 vs G5-S3;**(D)** MG5-S1 vs MG5-S2;**(E)** MG5-S1 vs MG5-S3; **(F)** MG5-S2 vs MG5-S3.

In the comparison among different materials and across various developmental stages, GO annotations were utilized to classify DEGs into biological processes, cellular components, and molecular functions (**Supplementary Table S5**). The top 20 GO terms were ranked based on Q-values < 0.05 for each of the six comparison groups. It was observed that compared to stage S1, significant GO terms were shared by G5 at stages S2 and S3, including ‘response to hydrogen peroxide’ (GO:0042542), ‘polymeric cytoskeletal fiber’ (GO:0099513), ‘cytoskeletal protein binding’ (GO:0008092), ‘external encapsulating structure organization’ (GO:0045229), ‘carbohydrate metabolic process’ (GO:0005975), ‘cell periphery’ (GO:0071944), and ‘plasma membrane’ (GO:0005886) (**Figures 4A, B**). Additionally, the three comparison groups of G5 exhibited shared GO terms, namely ‘external encapsulating structure organization’, ‘carbohydrate metabolic process’, and ‘cell periphery’ (**Figures 4A–C**). In contrast, MG5 showed significant GO terms at stages S2 and S3 compared to S1, such as ‘nucleosome’ (GO:0000786), ‘DNA packaging complex’ (GO:0044815), ‘nucleosome assembly’ (GO:0006334), ‘chromatin’ (GO:0000785), ‘cell periphery’, and ‘plasma membrane’ (**Figures 4D, E**). Furthermore, the three comparison groups of MG5 shared GO terms including ‘nucleosome’, ‘DNA packaging complex’, ‘cell periphery’, and ‘plasma membrane’ (**Figures 4D–F**). These distinctions in GO terms between the two materials may signify another pivotal factor contributing to developmental disparities.

#### 3.5.2 KEGG enrichment analysis

**Figure 5:**
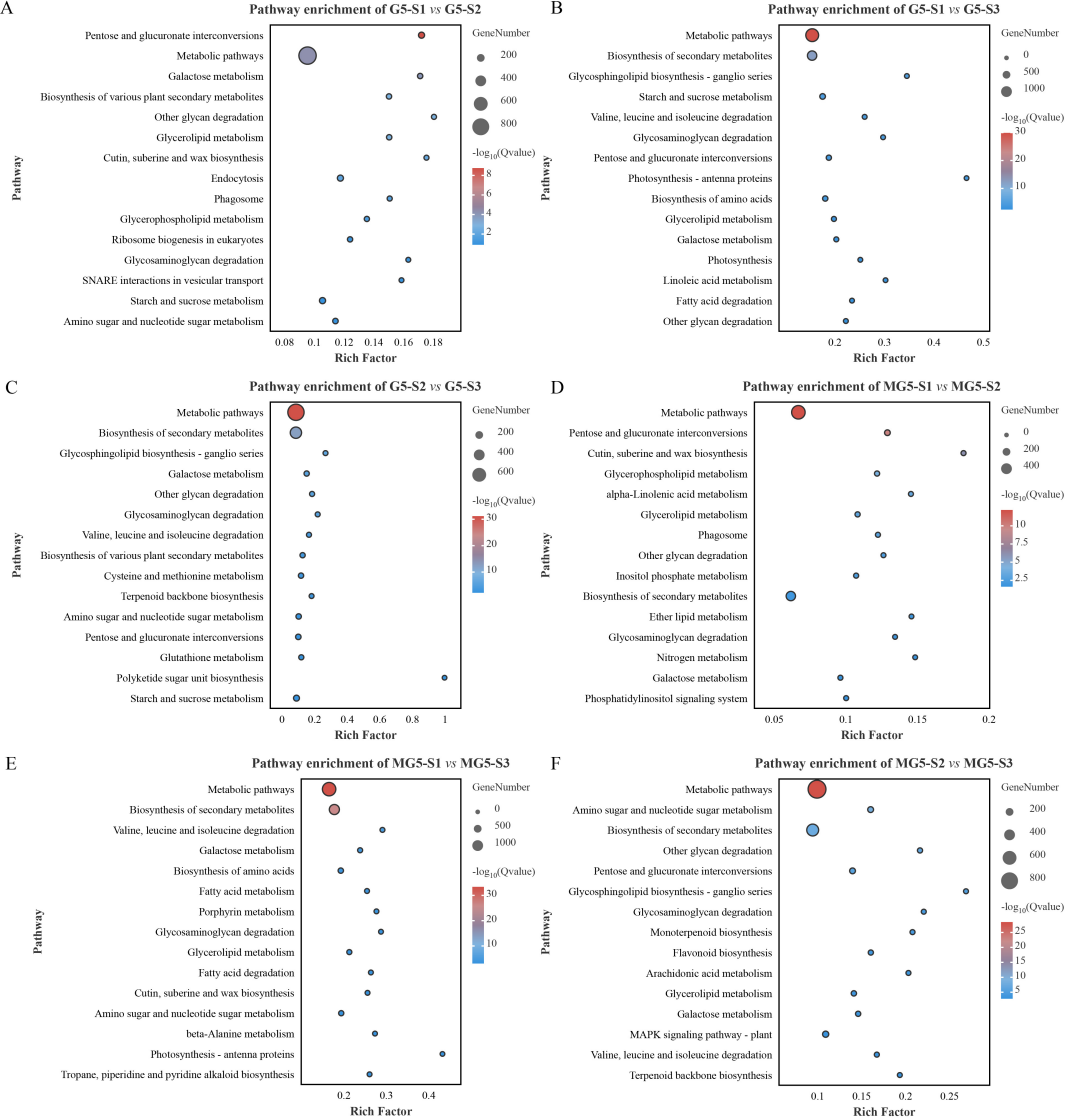
Kyoto Encyclopedia of Genes and Genomes (KEGG) enrichment analysis of the DEGs between MG5 and G5. **(A)** G5-S1 vs G5-S2; **(B) **G5-S1 vs G5-S3; **(C)** G5-S2 vs G5-S3; **(D)** MG5-S1 vs MG5-S2; **(E)** MG5-S1 vs MG5-S3; **(F)** MG5-S2 vs MG5-S3.

In order to further explore the metabolic pathways involving DEGs, KEGG analysis was conducted (**Figure 5**; **Supplementary Table S5**). Among the top 15 significantly enriched metabolic pathways, it was observed that pathways such as pentose and glucuronate interconversions, galactose metabolism, other glycan degradation, glycosaminogly can degradation, and starch and sucrose metabolism were significantly enriched in the comparison groups G5-S1 *vs* G5-S2, G5-S1 *vs* G5-S3 and G5-S2 *vs* G5-S3 (**Figures 5A–C**). Additionally, glycerolipid metabolism was significantly enriched only in the comparison groups G5-S1 *vs* G5-S2 and G5-S1 *vs* G5-S3 (**Figures 5A, B**). Metabolic pathways including glycerolipid metabolism, biosynthesis of secondary metabolites, glycosaminoglycan degradation, and galactose metabolism were predominantly enriched in the three comparison groups of MG5 (**Figures 5D–F**), while monoterpenoid biosynthesis, flavonoid biosynthesis, arachidonic acid metabolism, and MAPK signaling pathway-plant were enriched specifically in the comparison group MG5-S2 *vs* MG5-S3 (**Figure 5F**). Furthermore, glycerolipid metabolism was significantly enriched in all five comparison groups of both G5 and MG5 (except comparison group G5-S2 *vs* G5-S3), whereas galactose metabolism was significantly enriched in all six comparison groups of G5 and MG5, indicating their involvement throughout the development from floral bud to mature flower.

### 3.6 DEGs related to phytohormones and redox

**Figure 6:**
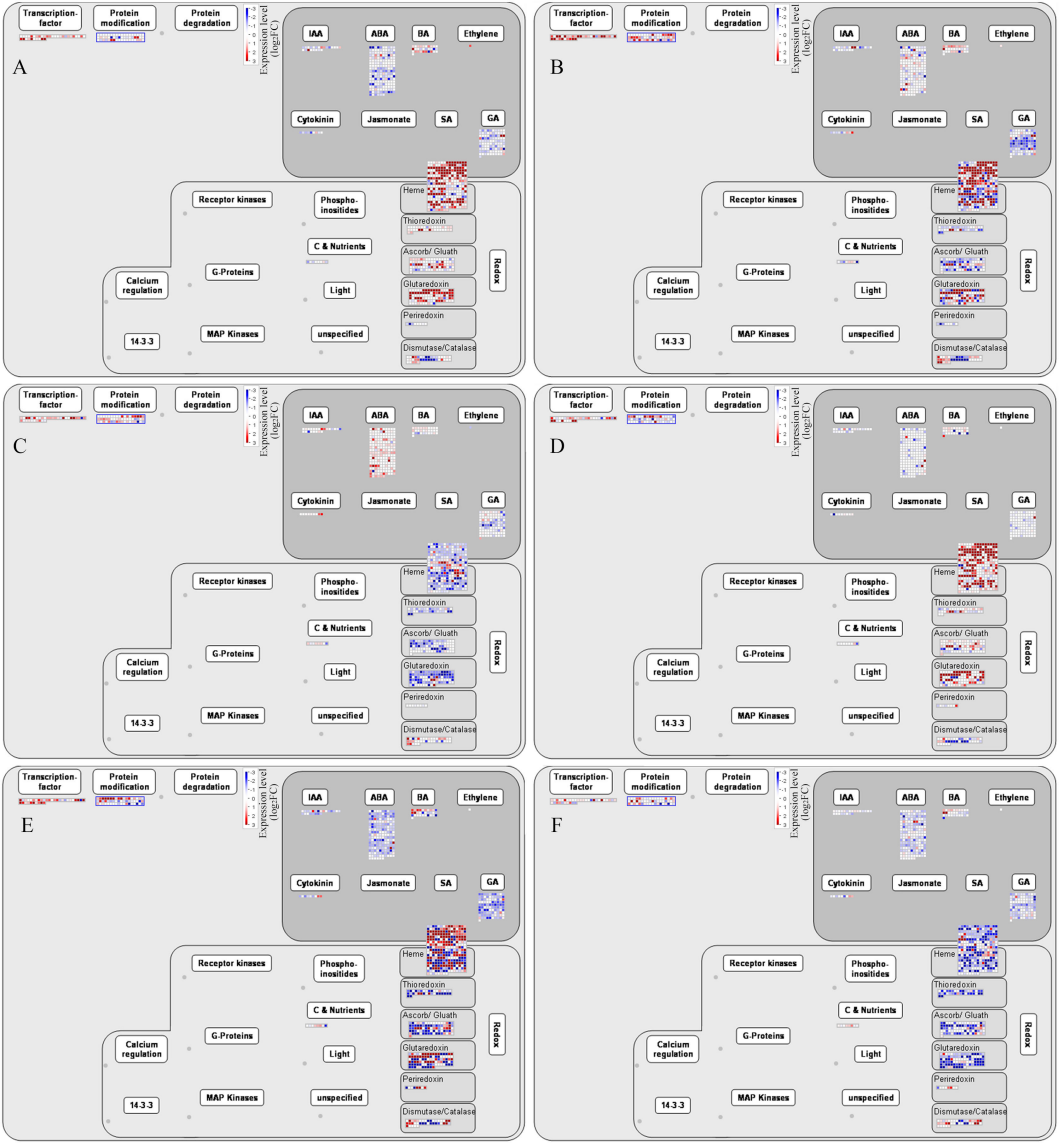
Mapman analysis of DEGs related to phytohormones and redox. **(A)** G5-S1 vs G5-S2, **(B)** G5-S1 vs G5-S3, **(C)** G5-S2 vs G5-S3, **(D)** MG5-S1 vs MG5-S2, **(E)** MG5-S1 vs MG5-S3, **(F)** MG5-S2 vs MG5-S3. **(E, F)** Number statistics of DEGs associated with phytohormones and redox.

To further analyze the regulatory pathways involved in the development of alfalfa flowers, we found through Mapman software that transcription factors, protein modifications, plant hormone signaling pathways, and redox pathways were all involved in the regulation of plant flower development (**Figure 6**). Among the six comparison groups of G5 and MG5, the hormone signaling pathways most prominently involved were ABA, followed by GA, brassinosteroids (BRs), IAA, and cytokinins (CK). The most enriched oxidative-reduction pathways included heme, followed by glutaredoxin, ascorb/gluath, dismutase/catalase, thioredoxins, and periredoxin (**Figures 6A–F**). Additionally, DEGs involved in regulating transcription factors, protein modification, heme, and glutaredoxin were mostly up-regulation in G5-S1 *vs* G5-S2, G5-S1 *vs* G5-S3, MG5-S1 *vs* MG5-S2, and MG5-S1 *vs* MG5-S3, whereas genes related to plant hormones and redox exhibited mostly down-regulated. Compared to stages S1 and S2, DEGs associated with plant hormones, redox, transcription factors, and protein modification at S2 and S3 stages of both materials displayed up-regulation or down-regulated expression. This suggested that these genes may modulate specific gene expression or intracellular substance levels through alterations in gene expression levels, thereby impacting cellular morphology and function.

### 3.7 Genes related to the regulation of flower development stages in alfalfa

**Figure 7:**
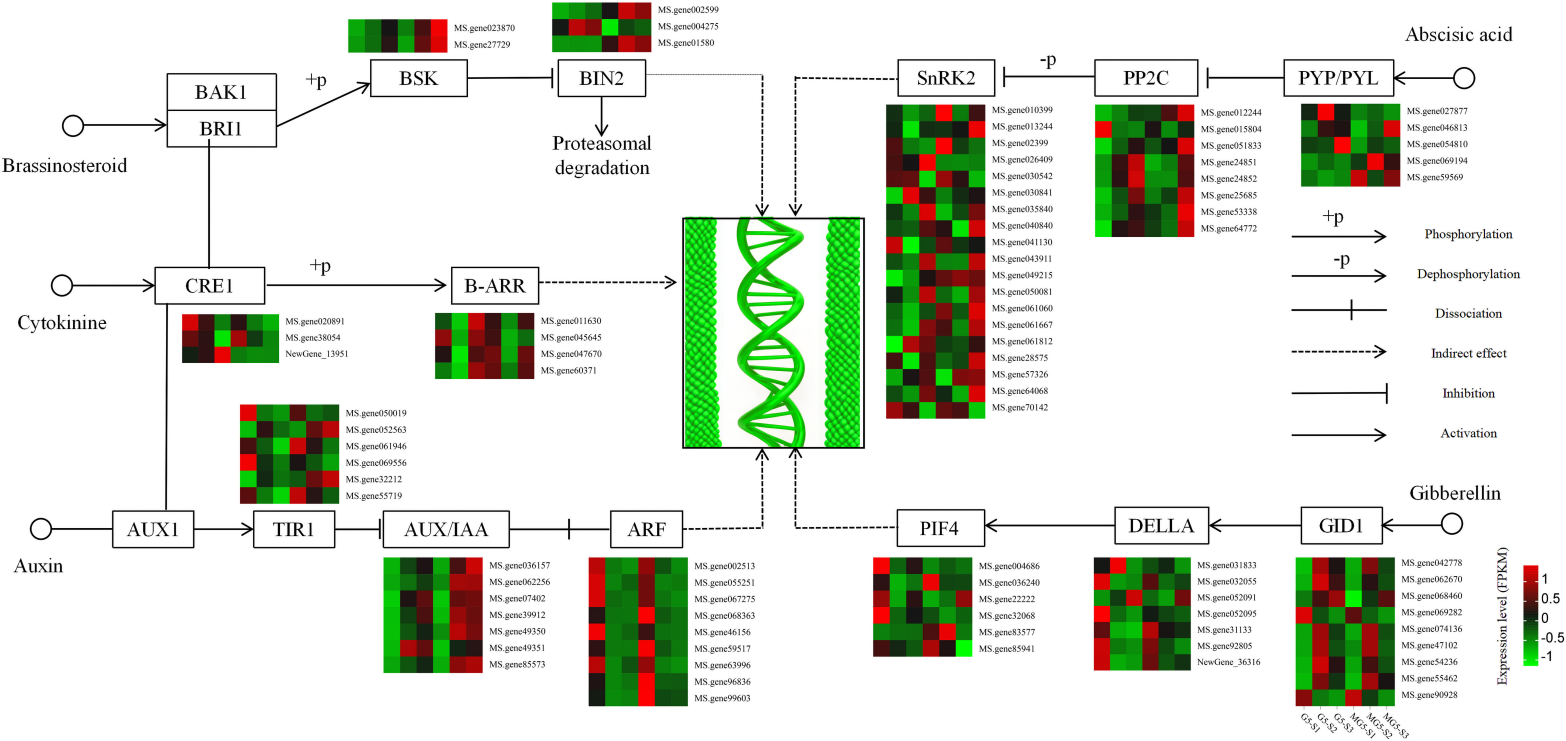
Heatmap analysis of the expression of DEGs involved in the plant hormone signaling transduction pathway.

Based on the analysis of the function and expression pattern of DEGs, a gene expression heatmap depicting the plant hormone signaling pathways associated with alfalfa flower development was constructed (**Figure 7**). In the BR pathway, the expression levels of BR signaling kinase (*BSK*) increased gradually with flower development. Compared to G5, *BSK* expression levels were consistently higher during the same stages than in MG5. Compared to stage S1, the gene encoding protein BR insensitive 2 (*BIN2*) showed significant increases at S2 and S3 stages of G5, while the other two *BIN2* genes exhibited significant increases at S2 and S3 stages of MG5. In the CK pathway, two cytokinin receptor, *Arabidopsis* histidine kinase (*CRE1*), were down-regulated in the comparisons (S1 *vs* S2) of G5 and MG5. One *CRE1* was up-regulation in the G5-S1 *vs* G5-S2 comparison. Additionally, two members of the two-component response regulators ARR-B family (*B-ARR*) genes were up-regulated in the G5-S1 *vs* G5-S2 comparison, with one *B-ARR* (Ms.gene052563) also showed up-regulation in MG5-S1 *vs* MG5-S2 and MG5-S1 *vs* MG5-S3. In the IAA pathway, the expression levels of six transport inhibitor response 1 (*TIR1*) genes varied across three stages of G5 and MG5, showing distinct changes in expression levels. Compared to stage S1, the expression levels of seven auxin responsive protein IAA (*AUX/IAA*) genes gradually increased during flower development stages in G5 and MG5. In contrast to *AUX/IAA* genes, the expression levels of nine auxin response factor (*ARF*) genes decreased gradually during flower development stages in G5 and MG5. In the ABA pathway, the expression levels of six ABA receptor PYR/PYL family genes differed across three stages of G5 and MG5. Compared to stage S1, the expression levels of protein phosphatase 2C (*PP2C*) genes significantly increased at stage S3, with three *PP2C* genes showed notable increases only at stage S3 of G5, suggesting that *PP2C* genes may primarily participate in alfalfa flower development from stage S2 to S3. Additionally, significant differences were observed in the expression levels of 19 serine/threonine protein kinase SRK2 (*SnRK2*) genes at various developmental stages between G5 and MG5 (**Supplementary Table S6**). Most *SnRK2* genes were up-regulation at stages S3 of G5 and MG5 compared to stage S1. Interestingly, we also found that the ABA content varied significantly between G5 and MG. Combined with previous reports of ABA regulation of flower development (Martignago et al., 2020), we inferred that ABA pathway-related genes primarily participated in regulating the transition from early to late flowering. In the GA pathway, compared to stage S1, expressions of seven gibberellin receptors *GID1* were up-regulation in the comparison groups G5-S1 *vs* G5-S2 and MG5-S1 *vs* MG5-S2, while the expression levels of the other two *GID1* genes notably decreased at S2 and S3 stages of G5 and MG5. The expression levels of five downstream genes of *GID1*, DELLA, were significantly reduced at S2 and S3 stages of G5 and MG5. The expression of downstream gene phytochrome interacting factor 4 (*PIF4*) exhibited varying degrees of changes in both materials. From the above analysis, it can be observed that genes associated with IAA, ABA, CK, GA, and BR signaling pathways in plant hormone signaling pathways participating in alfalfa flower development in distinct expression patterns.

### 3.8 DEGs encoding proteins interaction network analysis

**Figure 8:**
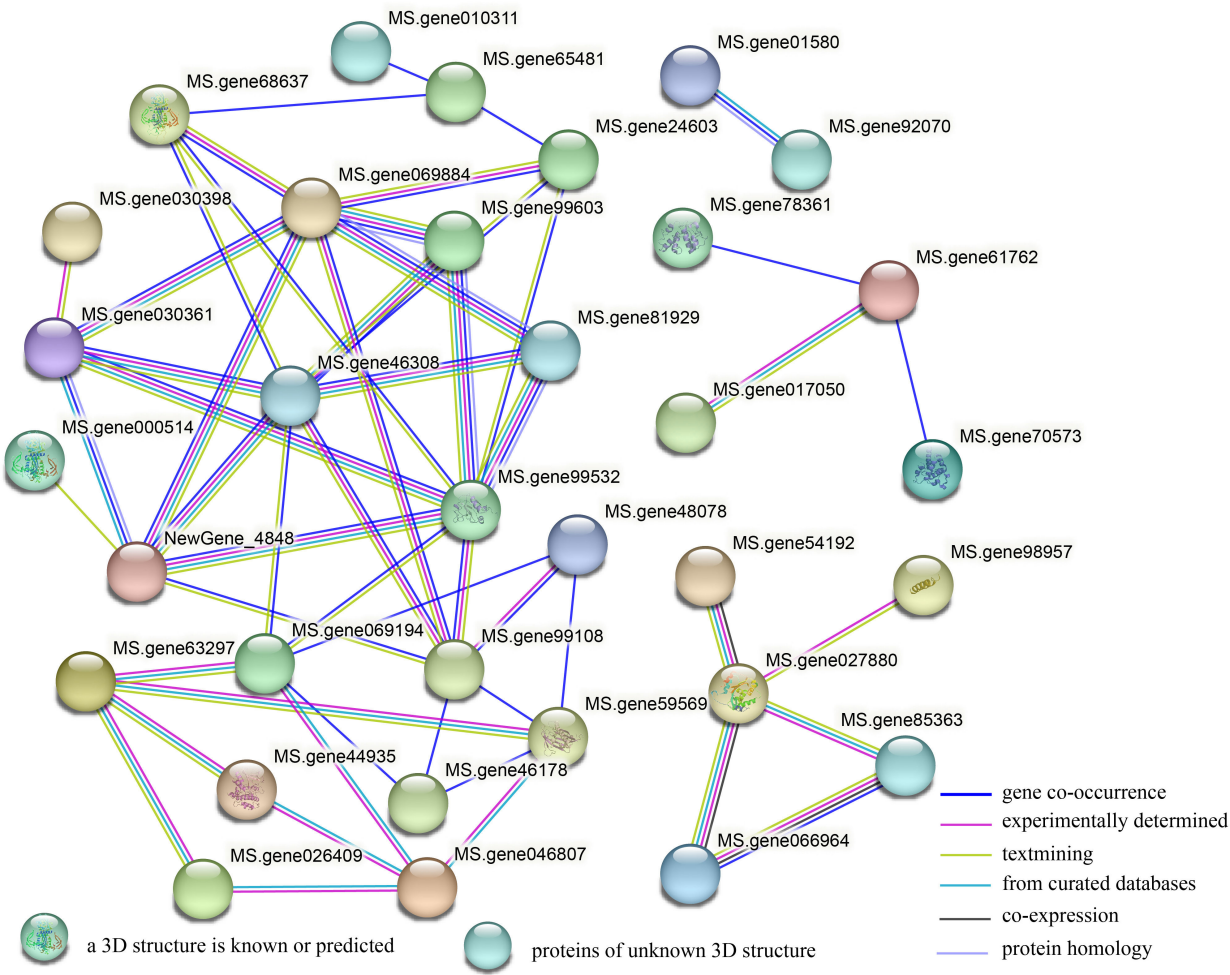
A protein interaction network of the DEGs encoding protein between G5 and MG5. The network was built using a String program with confidence greater than 0.4. The nodes represent the DEGs encoding protein, and the line color indicates the type of interaction evidence.

To determine the transmission mode of signals between genes related to plant hormone signal transduction, we conducted interaction analysis on DEGs encoding proteins in the plant hormone signal transduction pathway using a String database. It was found that 54 proteins had four groups of interactions (**Figure 8**). The first group contains only two proteins of the protein kinase family, mainly involved in the BR signaling pathway. The second group includes: transcription factor bZIP family, and transcription regulatory factor ABTB family members. The third group includes: transcription factor TIFY family, and Basic helix loop helix (BHLH) family transcription factor. The fourth group includes the largest number of proteins, mainly related genes involved in the regulation of auxin signal transduction and some genes related to other hormone signaling pathways. In addition, in the hub gene network, *Aux/IAAs* (Ms.gene46308, Ms.gene99532, Ms.gene069884), *ARF* (Ms. gene99108), ABA receptor PYR/PYL (Ms. gene069194), and *TIR1* (NewGene_4848) have the highest number of interacting proteins, with eight, eight, seven, seven, six, and six, respectively, indicating that these genes play a crucial role in plant hormone signal transduction.

### 3.9 Transcription factor analysis

**Figure 9:**
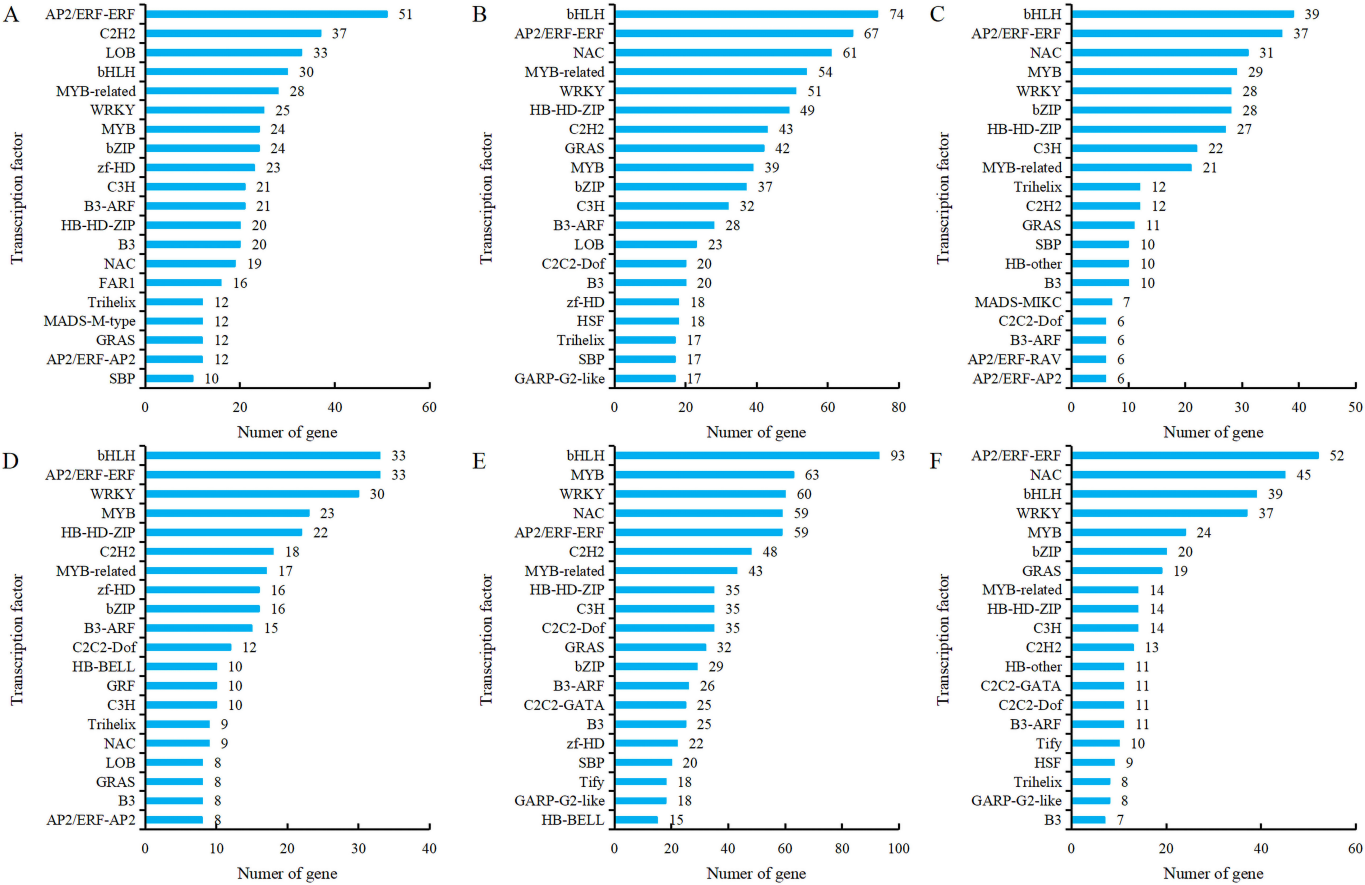
Analysis of the top 20 TFs with different expression between G5 and MG5. **(A)** G5-S1 *vs* G5-S2, **(B)** G5-S1 *vs* G5-S3, **(C)** G5-S2 *vs* G5-S3, **(D)** MG5-S1 *vs* MG5-S2, **(E)** MG5-S1 *vs* MG5-S3, **(F)** MG5-S2 *vs* MG5-S3.

Transcription factors (TFs), as key regulatory proteins mediating transcriptional regulation, play an important role in flower development. Transcription factor prediction was performed on all genes obtained from transcriptome sequencing, and it was found that 64 transcription factor families were identified in comparison groups G5-S1 *vs* G5-S2, G5-S1 *vs* G5-S3, G5-S2 *vs* G5-S3, MG5-S1 *vs* MG5-S2, MG5-S1 *vs* MG5-S3 and MG5-S2 *vs* MG5-S3. Among them, 38 transcription factor families were present in each comparison group, indicating that many transcription factors were involved in the complex regulation of alfalfa flower development. The most identified transcription factors in the comparison group G5-S1 *vs* G5-S2 were AP2/ERF-ERF, C2H2, and LOB (**Figure 9A**). In comparison groups G5-S1 *vs* G5-S3 and G5-S2 *vs* G5-S3, the most identified transcription factors were bHLH, AP2/ERF-ERF, and NAC (**Figures 9B, C**). In comparison group MG5-S1 *vs* MG5-S2, the most identified transcription factors were bHLH, AP2/ERF-ERF, and WRKY (**Figure 9D**), whereas MG5-S1 *vs* MG5-S3, the most identified transcription factors were bHLH, MYB, and WRKY (**Figure 9E**), and AP2/ERF-ERF, NAC, and bHLH were identified in MG5-S2 *vs* MG5-S3 (**Figure 9F**). Moreover, significant differences in the number of predicted transcription factors were evident across the six comparison groups. The comparison of the same stage and the same transcription factor showed that the comparison group G5-S1 *vs* G5-S2 had more DEGs than MG5-S1 *vs* MG5-S2, while MG5-S1 *vs* MG5-S3 and MG5-S2 *vs* MG5-S3 had more DEGs compared to G5-S1 *vs* G5-S3 and G5-S2 *vs* G5-S3, respectively.

### 3.10 qRT-PCR validation

Based on the plant hormone signaling pathway, we randomly selected 12 genes for qRT-PCR to verify the authenticity and reliability of the sequencing data (**Supplementary Figure S1**). The results indicate that both show a consistent trend of change. Compared to G5, PRP1C in MG5 was only up-regulated at the S1 phase, and the down-regulation trend at the S3 phase was not significant, which is different from the expression of RNA-seq. The expression of the remaining 11 DEGs was consistent with the RNA-seq results. Therefore, the transcriptome data obtained from RNA-seq analysis was true and reliable.

## 4 Discussion

RNA-seq sequencing technology was utilized to identify 31,207,730-41,989,022 alignment sequences from 18 alfalfa flower samples. Based on the reference genome alignment results, 23,897 new genes were identified, among which, 12,564 genes identified have complete functional annotations, providing new clues for exploring the regulation mechanism of flower development related genes in leguminous plants. The G5 and naturally occurring variant plant MG5 were selected as the subjects of this study. Compared to G5, MG5 exhibited typical characteristics of early flowering and increased flower production. A total of 547 DEGs were identified in the three stages of flower development between G5 and MG5, speculating that these genes play important functional roles in the flower development of alfalfa. By utilizing gene functional enrichment analysis, we identified two main metabolic pathways involved in the development of alfalfa flowers, redox and plant hormone signaling. And Some candidate genes for flower development were also identified (**Supplementary Table S7**). This provides a theoretical basis for the molecular mechanism analysis of subsequent leguminous plant flower development.

### 4.1 Regulation of redox process on the development of alfalfa flowers

Reactive oxygen species (ROS) play a crucial role in regulating many developmental processes, including aging, as well as plant responses to biotic and abiotic stress, particularly in binding to related reduction/oxidation (redox) controls. Glutathione (GSH), glutathione, and thioredox/oxidation (redox) play an irreplaceable role in plant growth and development (Ogawa et al., 2004; Xing et al., 2006). Previous studies have confirmed that ROS and GSH, together with nitric oxide (Airaki et al., 2011), are key regulatory factors in plant development (Considine and Foyer, 2014). Oxidation-reduction molecules such as glutathione and glutathione disulfides are particularly important in the development of plant flowers (Zechmann and Müller, 2010; Zechmann et al., 2011a, b; Traverso et al., 2013; Schippers et al., 2016). Specifically, the redox state of these glutathiones plays a crucial role in flower development and pollen vitality (García-Quirós et al., 2020).

There are various pathways through which ROS, glutathione, and sulfur oxygen reduction/oxidation regulate plant flower development. Different conclusions have been obtained in the research of different plants, which together constitute the complex mechanism of oxidation-reduction reactions regulating plant flower development. The interaction between glutathione and plant hormones revealed another mechanism of its role in flower development. For example, the absence of GSH can cause changes in auxin metabolism, thereby inhibiting pollen germination. This may be related to changes in ROS accumulation in reproductive tissues during pollen and pistil development, pollen tube germination, and pollen pistil interaction (Zafra et al., 2010). Glutathione S-transferase (GST) is a kind of multifunctional enzymatic antioxidant, and it is also the key enzyme of glutathione metabolic pathway. GST can catalyze the covalent binding of GSH with electrophilic substrate and transfer it to plastids or vacuoles, and catalyze H_2_O_2_ to convert GSH to oxidized glutathione (GSSG), which plays an important role in plant growth and development, such as flower development (Anik et al., 2024). Therefore, the interaction between GSH and other proteins forms a molecular network that plays an important role in plant stem tip meristem and early flower development by controlling the redox regulation of the plant cell cycle. Interestingly, the results of this study showed that in addition to flowering time, *GST* was also involved in the flower development of alfalfa. The results showed that in G5, 15 *GST* genes were identified at S2 compared with S1; Compared with S2 stage, 38 up-regulated and one down regulated *GST* were identified at S3 stage. Fourteen *GST* were identified in the comparison group MG5-S1 *vs* MG5-S2, and 48 *GST* were up-regulated and six GST were down regulated in the comparison group MG5-S2 *vs* MG5-S3. This result suggested that glutathione mainly played a role in the later stage of alfalfa development, mainly involved in the development of flowers at the early flower stage to late flower stage. In addition, we found that a large number of glutathione peroxidase genes were differentially expressed at different stages. Meanwhile, we found that a glutathione peroxidase (Ms.gene072788) was up-regulated in S1 *vs* S2 of the both materials, while down regulated in MG5-S2 *vs* MG5-S3. This stage specific expression pattern also indicated that different genes need to be activated at different stages of flower development. Therefore, the above analysis showed that these *GST* genes synergistically regulateed the steady state of redox system, thus regulating the development of alfalfa.

### 4.2 Regulation of plant hormone signal transduction on the development of alfalfa flowers

Plant hormones are involved in the entire process of plant growth and are crucial for plant development. In this study, it was found that genes related to the signal transduction pathways of common plant hormones (ABA, IAA, BR, CK, GA) were identified to be differentially expressed at the three stages of flower development in alfalfa. In the IAA pathway, the expression levels of seven *Aux/IAA* genes in G5 and MG5 gradually increased with the development of flowers, while the expression levels of nine *ARF* genes in G5 and MG5 decreased gradually with the development of flowers, indicating that the involvement of auxin was necessary to maintain sustained changes during the process of alfalfa flower development. A recent study found that the interaction between *Aux/IAA*, a plant auxin responsive gene, and *ARF* may play an important role in flower development through the auxin signaling pathway (Si et al., 2023), which also confirms our research.CK mainly regulates the activity of reproductive meristem tissue, flower organ size, and ovule formation, thereby regulating seed yield (Bartrina et al., 2011). This study found that the enriched *CRE1* and *B-ARR* genes in the CK pathway, as well as the enriched *AUX/IAA* and *TIR1* genes in the auxin pathway, were significantly up-regulated in the three stages of flower development. This suggests that CK may interact with IAA pathway related genes to regulate flower development in alfalfa. BR can promote filament elongation and anther development, and has a positive effect on regulating male flower fertility (Ye et al., 2010). Previous studies have reported that *Arabidopsis* BR deficient mutants exhibit inhibition in BR biosynthesis and signal transduction pathways, such as *bin2* exhibiting reduced fertility or male infertility, and a significant decrease in pollen grain count (Li et al., 2001). In this study, we found that the expression of two *BSK* genes were significantly up-regulated in the comparison group G5-S1 *vs* G5-S2, G5-S1 *vs* G5-S3, MG5-S1 *vs* MG5-S2 and MG5-S1 *vs* MG5-S3, but not significantly in S2 *vs* S3 of G5 and MG5. We also found that one *BIN2* gene was significantly increased at S2 and S3 of G5, and the other two *BIN2* genes were significantly increased at S2 and S3 of MG5. This indicated that the changes in BR during alfalfafa flower development were diverse and involve many genes. It was also closely related to the stage of flower development.

The differential expression of these genes jointly balances BR and regulates the process of flower development. There are research reports that *NnSnRK1* silenced lotus seedlings have strong flowering ability, with a 40% increase in flower to leaf ratio. The changes in ABA content are related to the expression of *NnSnRK1*. After ABA treatment, the expression level and protein kinase activity of *NnSnRK1* were significantly reduced. In addition, ABA treatment can enhance the phenotype of *NnSnRK1* silenced seedlings, while tungstate treatment can reverse its phenotype, indicating that ABA regulates plant flowering by controlling the expression of *NnSnRK1* (Cao et al., 2021). In this study, we found that the expression levels of 19 *SnRK2* genes were significantly different at different developmental stages of G5 and MG5. Compared to stage S1, most *SnRK2* genes were up-regulated at S3 stage of G5 and MG5, indicating that flower development has stage characteristics, and the development speed and organ types at different stages have significant differences. The multiple flowers are prominent features of MG5, and the strong correlation with the expression level of *SnRK2* gene is extremely interesting. Further in-depth analysis of the molecular mechanism of *SnRK*2 gene regulating flower development in alfalfa can be conducted in future research.

The loss of function of many components involved in GA biosynthesis and signal transduction leads to flowering defects (Wilson et al., 1992; Sun and Kamiya, 1994), which has been studied for over thirty years. *DELLA*, as a central inhibitory factor of the GA signaling pathway, has been shown to interact with the activity of many TFs to regulate flower development. We found that the expression levels of seven *GID1* were up-regulated in comparison group G5-S1 *vs* G5-S2 and MG5-S1 *vs* MG5-S2, while the expression levels of the other two *GID1* were significantly decreased at S2 and S3 stages. The expression levels of five *DELLA* were significantly decreased at S2 and S3 stages of G5 and MG5. This suggested that these genes related to GA were involved in the development of alfalfa. In addition, we also measured the content of various endogenous hormones in two materials and concluded that the premature peak of endogenous ZT and ABA content in MG5 material compared to G5 may be one of the reasons for its more number of flowers. It is interesting that ABA and CK related genes were up-regulated in MG5 in this study. We speculate that the differential expression of these genes and the significantly different levels of ABA and CK are one of the reasons why natural mutant material MG5 has a larger number of flowers.

### 4.3 Transcription factors involved in the regulation of alfalfa flower development

Some molecular genetic studies have demonstrated the important role of transcription factors in plant reproductive development, and we have also found that transcription factors are significantly enriched in the three key stages of alfalfa flower development. Among various transcription factor families, MYB, AP2, bHLH, C2C2, MADS box, NAC, bZIP, B3, and AUX/IAA play important roles in the regulation of alfalfa flower development, and members of these gene families are also involved in the flower development of other plants (Hennig et al., 2004; Sharma et al., 2012; Singh et al., 2013). Early studies have shown that the MYB family plays an important role in the development of anthers and pollen (Zhu et al., 2004; Li et al., 2010a). During flowering and development, bHLH binding to MYB and MADS box genes plays a crucial role in the sex determination of cabbage type spinach (*Spinacia oleracea*) (Liu et al., 2021). The key role of MADS-box transcription factors in coordinating the specification and development of floral organs has been well demonstrated in several studies (Ng and Yanofsky, 2001; Kater et al., 2006; Urbanus et al., 2010). MADS-box homologous genes involved in flower development, such as *AP1*, *AP3*, *PP3*, and *AG*, are preferentially expressed in the flowers of chickpea (*Cicer arietinum*) (Singh et al., 2013). This study also identified the differential expression of multiple homologous genes of MADS-box in two materials, and the protective effects of these genes in leguminous plants, including alfalfa, and their expression profiles indicate the existence of similar transcriptional regulatory networks during flower development in plants (Hecht et al., 2005; Jung et al., 2012). Aux/IAA protein mediates auxin response and participates in plant flower development by interacting with ARF transcription factors (Si et al., 2023). In this study, we found that *Aux/IAA* genes were identified in six comparison groups of the two materials, and more than 50% of B3 family members were its subfamily ARF. In addition, we conducted protein interaction analysis on the DEGs encoded proteins in plant hormone signaling pathways using the String database. The results showed that *Aux/IAAs* (Ms.gene46308, Ms.gene99532, Ms.gene069884) and ARF (Ms. gene99108) had the highest number of interacting proteins and belonged to the core gene. This fully indicates that the interaction between AUX/IAA family members and their downstream ARF family members were involved in the regulation of plant flower development through the IAA signaling pathway.

## 5 Conclusion

In summary, the results found that the redox pathway and plant hormone signaling pathway were mainly involved in the flower development of alfalfa. Further gene functional identification revealed that the *GST* homologous gene was up-regulated or down-regulated in the redox pathway. Plant hormone signaling pathways included down-regulation of ABA related gene *SnRK2*, as well as up-regulation of BR related genes *BSK*, GA related genes *GID1* and *DELLA*, and CK related gene *CRE1*. In addition, a large number of transcription factors including MYB, AP2, bHLH, C2C2, MADS box, NAC, bZIP, B3, and AUX/IAA were also differentially expressed during the development of alfalfa flowers. Taken discussed above, the differential expression of these genes and transcription factors was one of the main genetic factor involved in the development of alfalfa flowers.

## Data Availability

The data presented in the study are deposited in the NCBI's Short Read Archive (SRA) repository, accession number PRJNA1100750.
