# Supplementary material for: Analysis on morphological characteristics and identification of candidate genes during the flowering development of alfalfa
Source: Front Plant Sci. 2024 Aug 13;15:1426838. doi: 10.3389/fpls.2024.1426838 (PMC11347289; doi:10.3389/fpls.2024.1426838)
Supplement: Supplementary file 1 [file DataSheet1.docx]

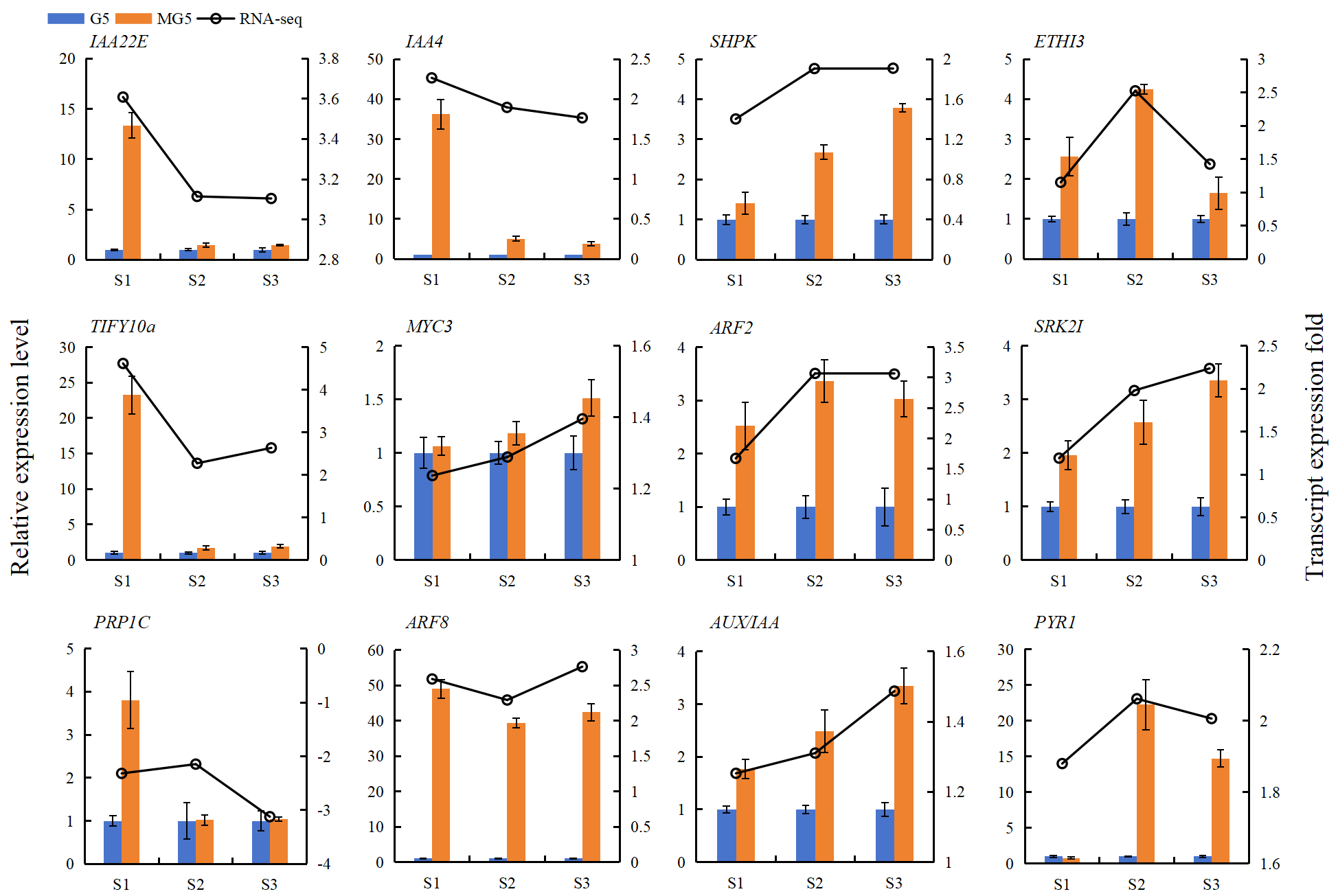


**Figure S1.** qRT-PCR analysis of changes in expression multiples of 12 selected genes. The bar chart with standard error represents the relative expression level determined by qRT-PCR (left y-axis), and the line chart with dots represents the change in transcript expression fold (log_2_FC) (right y-axis). Abbreviations: *IAA*, Auxin-induced protein; *SHPK*, shaggy-related protein kinase eta; *ETHI3*, ETHYLENE INSENSITIVE 3-like 3 protein; *TIFY10a*, protein TIFY 10a isoform X3; *MYC3*, transcription factor MYC3; *ARF*, auxin response factor; *SRK2I*, serine/threonine-protein kinase SRK2I; *PRP1C*, pathogenesis-related protein 1C; *PYP1*, abscisic acid receptor PYR1.
